# The progress of research on the application of redox nanomaterials in disease therapy

**DOI:** 10.3389/fchem.2023.1115440

**Published:** 2023-02-06

**Authors:** Xiaolu Shi, Ye Tian, Shaobo Zhai, Yang Liu, Shunli Chu, Zhengrong Xiong

**Affiliations:** ^1^ Department of Implantology, Hospital of Stomatology, Jilin University, Changchun, China; ^2^ Polymer Composites Engineering Laboratory, Changchun Institute of Applied Chemistry, Chinese Academy of Sciences (CAS), Changchun, China; ^3^ Department of Applied Chemistry, University of Science and Technology of China, Hefei, China

**Keywords:** redox, nanomaterials, reactive oxygen species, oxidative stress, tumor

## Abstract

Redox imbalance can trigger cell dysfunction and damage and plays a vital role in the origin and progression of many diseases. Maintaining the balance between oxidants and antioxidants *in vivo* is a complicated and arduous task, leading to ongoing research into the construction of redox nanomaterials. Nanodrug platforms with redox characteristics can not only reduce the adverse effects of oxidative stress on tissues by removing excess oxidants from the body but also have multienzyme-like activity, which can play a cytotoxic role in tumor tissues through the catalytic oxidation of their substrates to produce harmful reactive oxygen species such as hydroxyl radicals. In this review, various redox nanomaterials currently used in disease therapy are discussed, emphasizing the treatment methods and their applications in tumors and other human tissues. Finally, the limitations of the current clinical application of redox nanomaterials are considered.

## 1 Introduction

“Life is an instantaneous encounter of circulating matter and flowing energy.” This not only reveals the meaning of life but also explains the relationship between redox biology and metabolism (Korac et al., 2021). The balanced and harmonious function of the various systems and organs in the body is fundamental to a healthy life. However, due to the influence of various endogenous and exogenous factors, the body’s metabolic homeostasis can be disrupted and its redox balance could be altered. This imbalance has an important role in the development of numerous diseases, such as atherosclerosis, Alzheimer’s disease, and cancer (Sies and Jones, 2020; Forman and Zhang, 2021; Tasdogan et al., 2021).

Maintaining dynamic redox homeostasis is a constant challenge (Sies, 2021) as it involves complex chemical reactions of reactive species derived from oxygen and nitrogen. The imbalance between these oxidants and antioxidants can lead to oxidative stress, leading to abnormal cell function or death (Sies, 2017; Sies et al., 2017). The most common oxidants are reactive oxygen species (ROS), and they generally include hydrogen peroxide (H_2_O_2_), singlet oxygen (^1^O_2_), hydroxyl radicals (•OH), and superoxide anion radicals (O_2_^•-^). Among them, hydrogen peroxide is a stable non-radical ROS, which can be converted into other highly active ROS under catalytic conditions, such as ^1^O_2_, •OH, O_2_^•-^, or peroxynitrite (ONOO^−^) (Wu et al., 2019). Furthermore, it can be utilized as a substrate for O_2_ generation with specific enzymes to relieve tumor hypoxia (Yang et al., 2020a). An increase in ROS may lead to lipid peroxidation (LPO) and DNA and protein damage, which is referred to as oxidative stress (Dong et al., 2019). The main therapeutic measures against oxidative stress aim to prevent the production of oxidants that cause direct cellular damage, as this will inhibit the downstream signals of oxidants that lead to inflammation or cell death and increase antioxidant enzymes and their substrates (Forman and Zhang, 2021). However, the question of how to precisely regulate the levels of various oxidants or antioxidants in the tissue microenvironment to ensure therapeutic efficacy without causing significant cytotoxicity has become a focus of ongoing research.

Because of the remarkable breakthroughs in the field of nanotechnology, particularly nanochemistry and nanofabrication technologies, a variety of nanomaterials with unique redox-modulating properties have been prepared (Yang et al., 2019). Numerous experimental studies and clinical results have demonstrated that nanomaterials can act as antioxidants to scavenge various ROS or reactive nitrogen species using mechanisms similar to the body’s natural antioxidant mechanisms; they have also been described as having enzyme-mimetic activities (Korsvik et al., 2007; Pirmohamed et al., 2010). It is worth mentioning that in addition to mimicking superoxide dismutase to scavenge free radicals (Wang et al., 2020), redox nanomaterials can also mimic peroxidase activity by catalyzing ROS production through hydrogen peroxide, thus functioning as cytotoxic agents in tumor tissues (Dolmans et al., 2003; Li et al., 2008). Redox potential was found to be one of the main physiological differences between tumor and normal tissues, which facilitates the construction of redox-responsive nanodrug delivery systems (Mahdaviani et al., 2017). The rational design of redox-responsive nanomaterials for targeted therapies based on atopic changes in the redox environment in different tissues has also become a hot research topic.

**FIGURE 1:**
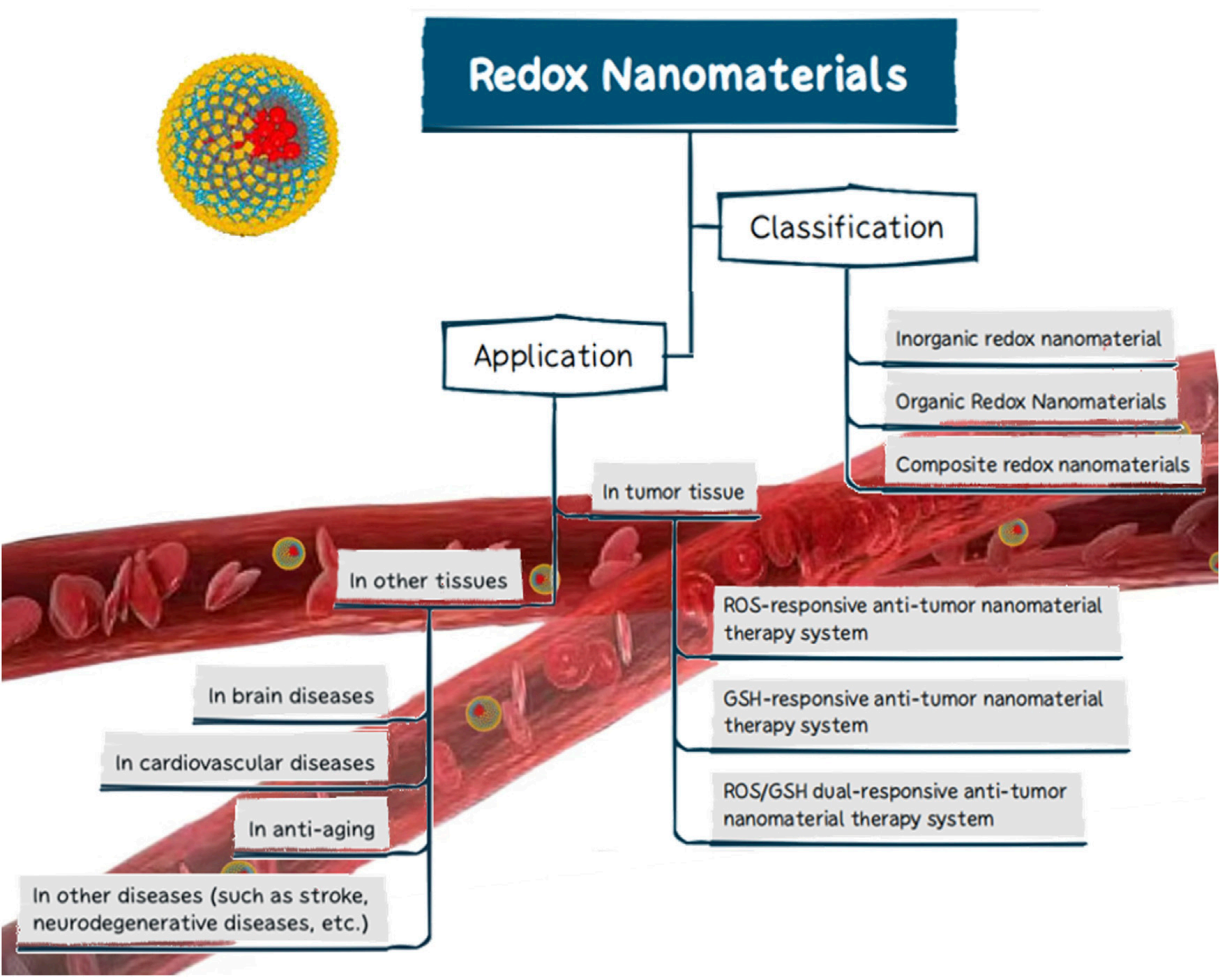
The classification and application of redox nanomaterials.

Currently, the use of redox nanomaterials in biomedicine has received increasing attention. While some studies have unilaterally reported the classification of redox nanomaterials or their applications in tumor therapy or other fields, few articles have integrated the existing data to provide a comprehensive summary. It may thus be difficult to fully understand the applicability of redox nanomaterials in the treatment of various diseases. In this review, we briefly summarized the redox nanomaterials that have been or are projected to be used across a wide range of biomedical applications, focusing on the different material types and their redox properties. We have also described the therapeutic pathways underlying the use of redox nanomaterials in tumor tissue and some of the research advances in other human tissue applications (Figure 1). Finally, we present a short summary of redox nanomaterials, followed by a brief analysis of the limitations of these nanomaterials in clinical applications and an outlook on their future development.

## 2 Classification of redox nanomaterials

In recent years, efforts have focused on the exploitation of sophisticated and effective nanomaterials that can be transported toward specific tissues with accurate release using spatial and temporal controls (Wolfram and Ferrari, 2019). Sustained release and site-specific drug delivery prevent premature drug release, thereby reducing the toxicity of nanomedicines to normal tissues. Demand for controlled drug release has fueled the rapid development of stimulus-responsive nanomaterials (Liu et al., 2016a; Liang et al., 2020). Redox-responsive drug delivery nanoplatforms not only overcome the delivery and pharmacokinetics deficiencies found in conventional drug deliveries but also elicit site-specific delivery properties, which help achieve superior therapeutic effects while reducing the biological toxicities of nanomedicines. In addition to responding to redox stimuli, it has been shown that nanomaterials with redox characteristics have promising antioxidant activities; their ability to scavenge ROS is of interest (Rzigalinski et al., 2006). However, the redox properties of nanomaterials can also promote the generation of ROS, leading to oxidative stress and causing tissue or cellular damage (Li et al., 2008); this property has been widely researched and applied in tumor therapy. In this review, they are uniformly referred to as redox nanomaterials. Depending on their chemical composition, redox nanomaterials are mainly classified as inorganic, organic, or composite (Table 1).

**TABLE 1:** Summary of common redox nanomaterials.

Classification	Nanomaterials	Redox sensitive moiety	Evaluation	Ref
Inorganic nanomaterials	AuNC-HA-PROT	AuNC	The study showed the nanomaterials had excellent and selective killing activity toward MDA-MB-231 cells by the light-induced toxic ^1^O_2_ produced by AuNC and the specific binding ability of HA to CD44	Xia et al. (2019)
	P@Pt@Cu	Pt@Cu	The study showed P@Pt@Cu presented excellent ROS scavenging ability by the enzyme-mimicking properties like superoxide dismutase, catalase, and glutathione peroxidase, leading to excellent antioxidant and anti-inflammatory activities in H_2_O_2_ stimulated chondrocytes and OA joints	Chen et al. (2022)
	Fe_3_O_4_-PLGA-Ce6	Fe_3_O_4_ and Ce6	The study showed Fe^2+^/Fe^3+^ and Ce6 could be released from Fe_3_O_4_-PLGA-Ce6 in the acidic TME. Then, the Fenton reaction between the released Fe^2+^/Fe^3+^and intracellular excess H_2_O_2_ could produce hydroxyl radicals (•OH) and induce tumor cell ferroptosis. The released Ce6 could increase the generation and accumulation of ROS under laser irradiation to offer photodynamic therapy, which can boost ferroptosis in 4T1 cells	Chen et al. (2021b)
	EGCNPs	Nanoceria	The study showed EGCNPs could enter the human lens epithelial cells to protect them from oxidative stress induced by hydrogen peroxide, improve the ratio between reduced and oxidised glutathione (GSH/GSSG) and provide a significant protection against the glycation of lens proteins	Hanafy et al. (2021)
	MSNs-SS-siRNA@Dox	-SS-	The study showed the disulfide linkages in MSNs-SS-siRNA@Dox could target intracellular GSH, and subsequent exposure to intracellular GSH levels led to their cleavage and the release of Dox and siRNA. Compared with free Dox, MSNs-SS-siRNA produced significant accumulation of Dox and exhibited satisfactory cytotoxicity by inducing apoptosis in MCF-7 cells	Zhao et al. (2017)
	NGO-SS-HA-Gef	-SS-	The study showed the target-specific binding of NGO-SS-HA to cancer cells by the cleavage of disulfide linkages significantly enhanced the abilities of gefitinib-loaded GO nanosheets to induce cell apoptosis, suppress cell proliferation, and inhibit tumor growth in lung cancer cell-bearing mice	Liu et al. (2018)
Organic nanomaterials	HA (HECS-ss-OA)/GA	-SS-	The study showed HA (HECS-ss-OA)/GA exhibited the highest apoptosis induction and cytotoxicity compared with the non-sensitive (HA (HECS-cc-OA)/GA) and HA un-coated (HECS-ss- OA/GA) controls against A549 NSCLC (non-small-cell lung cancer) model by the HA-receptor mediated cellular uptake and the disulfide linkages mediated burst drug release in highly reducing cytosol both *in vitro* and *in vivo*	Xu et al. (2019)
	IR/SS30-LP	-SS-	The study showed the irinotecan (IR) loaded liposomes based on disulfide phosphatidylcholine (SS-PC), IR/SS30-LP, could be triggered by GSH, leading to structural collapse and disintegrate to release IR, so it had superior pharmacokinetic and antitumor efficacy compared to free irinotecan and traditional irinotecan liposome	Wang et al. (2021)
Organic-inorganic hybrid nanomaterials	Gd-FLPNPs	-SS-	The study showed Gd-FLPNPs (a new kind of Gd complex and folate-coated redox-sensitive lipid-polymer hybrid nanoparticles) could release Dox faster, which was related to the cleavage of the disulfide bond of mPEG-S-S-C16 in the reducing environment, and enhance cell uptake *in vitro*, and exhibit better antitumor effect both *in vitro* and *in vivo*	Wu et al. (2017)
	{CuL-[AlOH]_2_}_n_	Cu^2+^ and porphyrin	The study showed Cu^2+^, the active center of MOF-2, could specifically bind and absorb GSH, thus directly decreasing the intracellular GSH concentration and increasing the ROS level, and the porphyrin ligand could generate abundant ROS under light irradiation. The two actions of MOF-2 could synergistically increase ROS concentration and accelerate apoptosis, thereby enhancing the effect of PDT.	Zhang et al. (2018)

### 2.1 Inorganic redox nanomaterials

Redox-responsive nanosystems based on inorganic nanocarriers are of great interest due to their unique physicochemical properties, such as robustness, cost-effectiveness, stability, and facile synthesis/modification (Mollazadeh et al., 2021). The production processes for inorganic nanoparticles can be scaled up as their size, shape, and surface functionalization can be easily controlled (Han et al., 2017). Controlled drug release can be achieved by introducing reduction- or oxidation-responsive bonds into inorganic nanomaterials (Han et al., 2017). The most widely used inorganic redox nanomaterials include metals and metal oxides, carbon groups, and mesoporous silica nanomaterials.

#### 2.1.1 Metal and metal oxide nanomaterials

**FIGURE 2:**
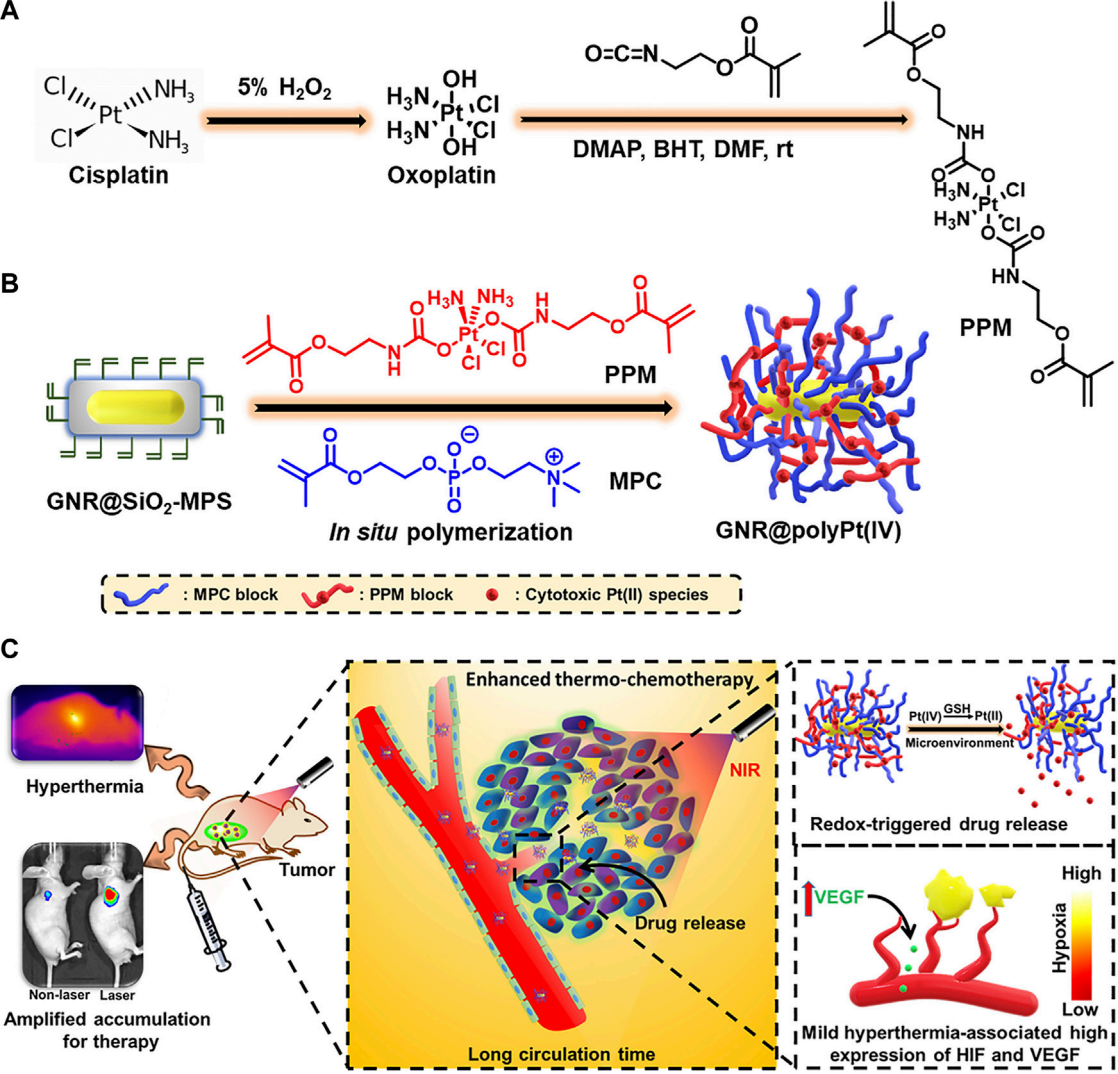
Illustration of the redox-responsive, *in situ*, polymerized GNR@polyPt (IV) for enhanced thermo-chemotherapy. **(A)** The synthesis of Pt (IV)-based prodrug monomer. **(B)** The preparation of GNR@polyPt (IV). **(C)** Schematic illustration of GNR@polyPt (IV) with redox-triggered drug release and mild-hyperthermia-amplified tumor penetration for enhanced thermo-chemotherapy of cancer. Reproduced with permission from (Guo et al., 2021) Copyright (2020) Elsevier.

Precious metal nanomaterials such as gold-, silver-, and platinum-based nanomaterials have great appeal in many nanomedicine applications due to their unique properties, including their excellent optical properties, good biocompatibility, and excellent chemical stability (Myroshnychenko et al., 2008; Cobley et al., 2011). While gold nanoparticles (AuNPs) are not generally considered to be redox-active, they can trigger other interactions that may lead to several biological redox reactions in biological systems (Sims et al., 2017). As an ideal platform for electrochemical biosensors, AuNPs are often used for the sensitive detection and quantification of ROS, such as H_2_O_2_ and O_2_^•-^(Guo and Dong, 2009; Sasidharan and Monteiro-Riviere, 2015). In recent years, the effects of oxidative stress due to the promotion of ROS production by AuNPs have been extensively studied for applications in photodynamic or photothermal therapies (Siddiqi et al., 2012). Khaing Oo et al. (2012) demonstrated the role of AuNPs in promoting ROS formation, which helped improve the efficacy of photodynamic therapy. In addition, AuNPs are susceptible to surface modifications by redox-responsive chemical bonds or groups, which can be ruptured in response to reducible constituents, such as GSH or photothermal reactions. Guo et al. (2021) successfully designed a redox-responsive nanoplatform (GNR@polyPt (IV)) with *in situ* polymerized polyplatinum (IV)-coated gold nanorods which can amplify tumor accumulation using mild hyperthermia for enhanced thermo-chemotherapy (Figure 2). In addition, Pt (IV) bis-urethanethyl methacrylate prodrug monomers were polymerized into redox-responsive polymers due to their symmetrical axial bisurethanethyl methacrylate ligands, thus endowing GNR@polyPt (IV) with redox-responsive properties.

Metal oxide nanomaterials such as iron oxide nanoparticles have become some of the most researched and versatile nanomaterials in biomedicine (Laurent et al., 2008). Iron oxide nanoparticles are widely used due to their magnetic quality in targeted drug delivery (Zhang et al., 2013), magnetic resonance imaging (Cheng et al., 2015a), tumor hyperthermia (Tabatabaei et al., 2015), and magnetically-assisted cell transfection (Jenkins et al., 2011) because of their superparamagnetism. Both Fe^2+^ and Fe^3+^ ions in iron oxide can catalyze a series of ROS-generating reactions, particularly the Fenton and Haber-Weiss reactions (Winterbourn, 1995). Furthermore, when the pH of the environment is low, iron oxide nanoparticles have peroxidase-like activity, which can catalyze the oxidation of peroxidase substrates to generate ROS, making them highly advantageous in tumor therapy (Gao et al., 2007). Iron oxide nanoparticles can also synergistically perform targeted drug delivery and controlled drug release by combining with drug carriers containing redox-sensitive bonds (e.g., disulfide bonds).

In addition to iron oxide nanoparticles, the redox mimetic antioxidant peculiarities of nanoceria have been effective in the treatment of numerous diseases caused by ROS or reactive nitrogen species (Lord et al., 2021). Nanoceria, a rare Earth metal oxide with a fluorite structure, has received attention due to its ability to mimic the activities of antioxidant enzymes, such as superoxide dismutase (SOD) and catalase (CAT) (Lord et al., 2021). Nanoceria can not only exhibit catalytic activity by switching the oxidation states between Ce^3+^ and Ce^4+^ (Karakoti et al., 2007), but also form oxygen vacancies to create oxygen defects in their fluorite crystalline lattice structure (Korsvik et al., 2007), thereby acting as a free radical scavenger in the physiological environment (Hirst et al., 2013).

In recent years, manganese dioxide (MnO_2_) and its nanocomposites, a redox-active transition metal dioxide nanomaterial, have been shown to be advantageous in cancer therapy due to their unique structural and physicochemical properties (Wen et al., 2020). MnO_2_ can be rapidly reduced to Mn^2+^ by some reducing substances through redox reactions as the valence state of Mn^4+^ in MnO_2_ is the intermediate valence, which endows MnO_2_ with strong catalytic activity and oxidation ability. In addition, MnO_2_ nanoparticles can trigger disproportionate intracellular H_2_O_2_, promoting ROS production and enhancing various ROS-based tumor therapies. Metal oxide nanomaterials, such as titanium oxide and zinc oxide, have redox properties and are often used in synergy to regulate redox homeostasis in different tissues to maximize their therapeutic effects.

#### 2.1.2 Carbon nanomaterials

With the increasing connection between nanomaterials and biomedicine, carbon nanomaterials, including graphene (Wen et al., 2012), fullerene (Shi et al., 2013), and carbon nanotubes (Wu et al., 2014), have been widely used as nanocarriers for targeted therapies due to their versatile functionalization chemistry, unique physicochemical properties, and significant specific surface area (Han et al., 2017). Carbon nanomaterials have a unique sp^2^ structure and hydrophobic nature, which means they can interact with drugs *via* either covalent conjugation, non-covalent absorption, hydrophobic interactions, or π-π stacking (Chen et al., 2015). In addition, their unique structural characteristics allow them to emit Raman vibration signals, which can be used to monitor distribution, metabolism, and excretion *in vivo*, such as tracking the release dynamics of redox nanomedicines (Chen et al., 2014).

Compared to graphene, graphene derivatives such as graphene oxide (GO) and reduced graphene oxide (rGO) are more widely studied and used in nanomedicine due to their better solubility and dispersibility. GO and rGO are often used as antibacterial/antimicrobial agents due to their peroxidase-like redox ability (Song et al., 2010; Li et al., 2016b). The basal planes and edges of GO have a high density of oxygen defect sites. This allows oxygen molecules to adsorb to the defective site and then be converted to ROS through electron transfer, resulting in the inactivation of bacteria (Perreault et al., 2015). Numerous data have also shown the feasibility and practicality of using GO-based nanomaterials to inactivate various bacteria (Gurunathan et al., 2012; Nanda et al., 2015).

Unlike GO, which induces oxidative stress by promoting ROS production, fullerene (C_60_) is commonly used as an antioxidant to protect neural tissues as it scavenges excess free radicals (Dugan et al., 1997). In addition, fullerene derivatives have been found to protect cells from ROS-induced oxidative damage, stabilize the mitochondrial membrane potential, reduce intracellular ROS production, and have a significant effect on delaying cellular senescence (Ali et al., 2004; Yin et al., 2009). Other carbon nanomaterials can also play a redox-modulating role by linking to structures or groups with redox properties. For example, the covalent modification of carbon nanotubes is often used as a means by which to modulate ROS production (Sims et al., 2017).

#### 2.1.3 Mesoporous silica nanomaterials

As a new inorganic nanomaterial, mesoporous silica nanoparticles (MSN) have good biocompatibility (Asefa and Tao, 2012), highly ordered mesopores, tunable pore sizes and volumes, and high specific surface areas (Chen et al., 2013). The MSN was readily functionalized on the surface, allowing for the introduction of cleavable redox-responsive bonds on their siloxane backbone to give them redox properties, which is why they are widely used in pharmaceutical formulations (Ma et al., 2014; Saikia et al., 2016). Zhao et al. (2017) designed a novel redox-responsive nanosystem (MSNs-SS-siRNA@Dox) loaded with anticancer drugs and siRNAs for targeted intracellular drug release and synergistic therapy. The siRNA plays a gatekeeper role by attaching to the MSN surface through a redox-responsive cleavable disulfide bond sensitive to glutathione (GSH). When MSNs-SS-siRNA@Dox is exposed to high levels of GSH in the tumor tissue, the disulfide bond is cleaved, leading to the rapid release of Dox and siRNA, resulting in a satisfactory therapeutic effect as it inhibits tumor growth *in vivo*.

#### 2.1.4 Other inorganic redox nanomaterials

In addition to the inorganic redox nanomaterials mentioned above, selenium nanoparticles (SeNPs) also have a wide range of biomedical applications due to their redox-regulating properties. In the human body, selenium is present as selenocysteine in various antioxidant enzymes, such as glutathione peroxidase, thioredoxin reductase, and selenoprotein P. As the redox center for these enzymes, selenium is vital in maintaining cellular redox homeostasis (Khurana et al., 2019). The concentration, chemical form, redox potential, and therapeutic mode are all critical determinants of the therapeutic activity of selenium compounds. For example, at low doses, it is an antioxidant, while at high doses, it becomes a pro-oxidant (Weekley and Harris, 2013; Ahmad et al., 2015). Both selenium nanoparticles and selenium-containing nano-polymer materials demonstrated obvious redox regulation properties and achieved an ideal curative effect in related disease models, which has been proved in previous research (Huang et al., 2020; Gangadevi et al., 2021). In addition, inorganic nanomaterials such as mesoporous hydroxyapatite nanoparticles (Li et al., 2014) and layered double hydroxides (Rodriguez et al., 2014) have also been used to construct redox-responsive nanomedicines (Han et al., 2017). While each of these inorganic redox nanomaterials has its pathway or mechanism to exert redox regulation, there is a growing interest in combining them to design multifunctional redox-responsive nanosystems.

### 2.2 Organic redox nanomaterials

Although inorganic nanomaterials are easy to prepare and are highly modifiable (Jaque et al., 2014), their clinical applications are hampered by their potential cytotoxicity due to their poor biodegradability (Kowada et al., 2015). In contrast, organic nanomaterials tend to be more biodegradable and biocompatible (Guha et al., 2015; Liu et al., 2019). In previous decades, organic nanomaterials based on liposomes, polymeric micelles, nanogels, dendrimers, and polymers have been widely used to develop redox-sensitive nanopharmaceutical carriers. Some of these nanocarriers, such as liposomes, have been approved by the US Food and Drug Administration and are already in clinical use (Han et al., 2017).

Liposomes are composed of phospholipid membranes that mimic natural membranes and have features such as easy development with controlled size distributions and no innate toxicity; they are often used to construct nanodrug carriers (Lee and Thompson, 2017). Liposomal nanoparticles are often cross-linked with redox-responsive agents such as disulfide bonds (-S-S-), diselenide bonds (-Se-Se-), thioether bonds (-S-), or thiol groups (-SH) to target redox-responsive sites (Zhou et al., 2018; Ling et al., 2019; Shi et al., 2020). In response to tissue-specific stimuli, redox-responsive liposomal nanoparticles promote drug release through disulfide bond breaking or the destabilization of liposomal membranes.

**FIGURE 3:**
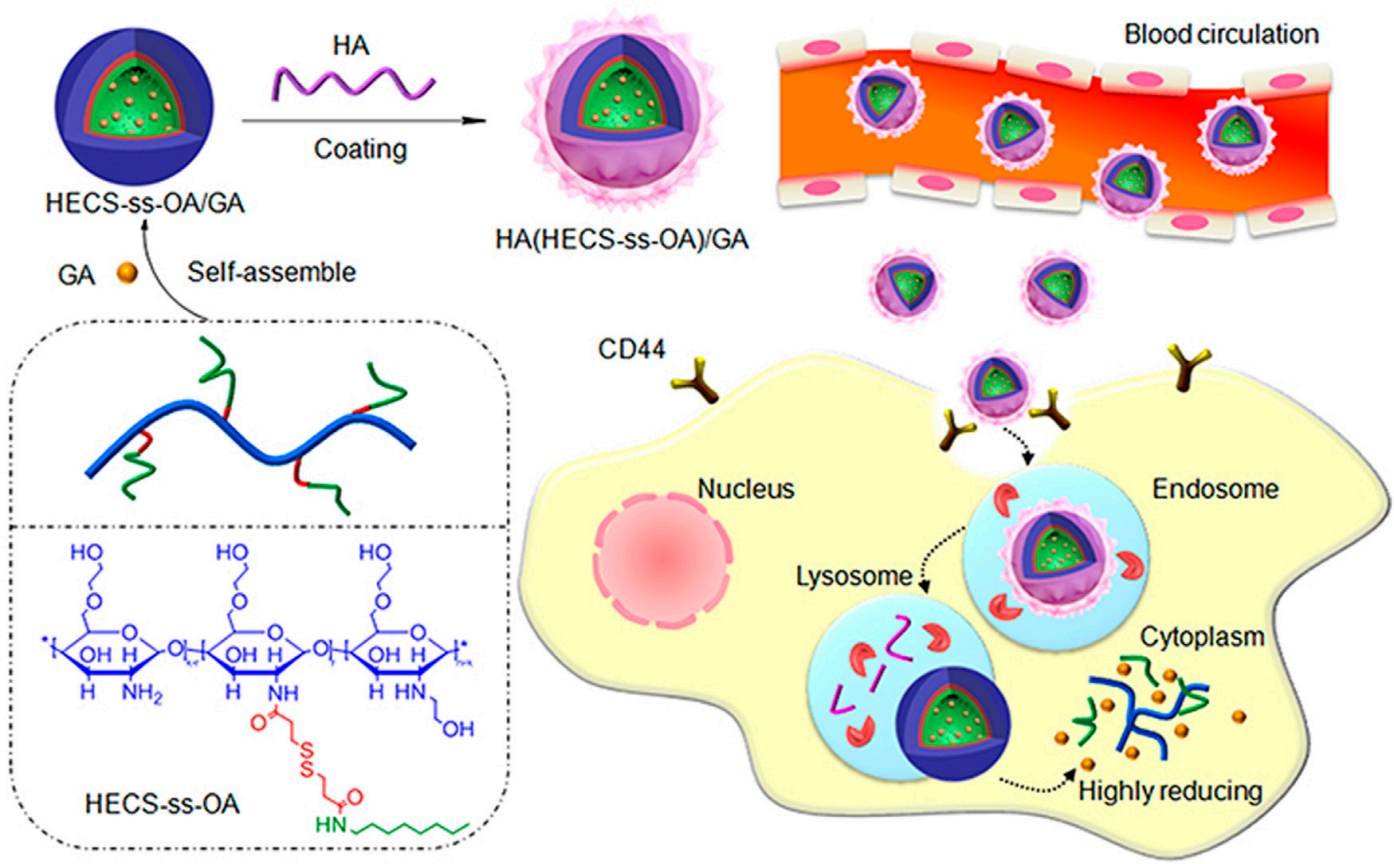
The structure of HA (HECS-ss-OA)/GA and the illustration of tumor cytoplasm-selective rapid GA delivery by the HA-coated redox-sensitive HA (HECS-ss-OA)/GA nanoparticles. GA was solubilized into the inner core of HECS-ss-OA micelles, while HA was employed to coat outside HECS-ss-OA/GA. HECS-ss-OA, redox-sensitive O, N-hydroxyethyl chitosan−octylamine conjugates; GA, gambogic acid; HA, hyaluronic acid. Reproduced with permission from (Xu et al., 2019) Copyright (2019) Dove Medical Press Limited.

Polymeric micelle nanoparticles are usually composed of amphiphilic copolymers, whose hydrophobic parts self-assemble in an aqueous solution into a core surrounded by the hydrophilic part as a shell, forming a typical core-shell structure that contributes to creating a drug loading carrier (Mollazadeh et al., 2021). Like liposomal nanoparticles, polymeric micelles are often endowed with redox responsiveness through the modification of disulfide bonds (Li et al., 2016a; Sun et al., 2018b), thereby improving the targeting selectivity and reduction of undesirable side effects (Davis et al., 2008). For example, the structure and treatment of a redox polymeric micelle nanoparticles designed by Xu et al. (2019) are shown in Figure 3.

Among the many organic nanocarriers, nanosized hydrogels offer a controlled-release pattern of drugs in targeted tissues and passive targeting (Chen et al., 2017; Ghorbani and Hamishehkar, 2019). The flexibility and softness of nanogels allow increased sensitivity to different stimuli and easier penetration into tissues (Ding et al., 2018; Mohtashamian et al., 2018). Redox-active units, such as disulfide, ditellurium, and diselenide bonds (Kumar et al., 2019), are attached to the network or in-between drugs and combined using other inorganic redox nanoparticles with nanogels to construct redox nanogel platforms, which have emerged as promising candidates as efficient redox-responsive drug delivery systems (Yin et al., 2020; Huang et al., 2021a; Zhu et al., 2021; Liu et al., 2023).

In contrast to the redox properties of most inorganic redox nanomaterials, organic redox nanomaterials are also often redox-responsive due to their attachments to various chemical bonds or groups with redox properties, such as dendrimers (Zhao et al., 2011) and various polymers (Hsu and Almutairi, 2021). Given the limitations of single-nature materials, composite nanomaterials containing organic and inorganic redox nanomaterials have emerged as a new generation of promising redox nanoplatforms.

### 2.3 Composite redox nanomaterials

Composite redox-responsive nanomaterials that integrate inorganic and organic redox nanomaterials combine their respective strengths and improve their physicochemical properties and often exhibit synergistic effects (Li et al., 2019a; Hao et al., 2020). Numerous experimental data and studies have demonstrated the high potential of composite nanomaterials in passing pharmacokinetic barriers, enhancing drug bioavailability, circulation time, drug release, and targeted delivery (Mollazadeh et al., 2021).

**FIGURE 4:**
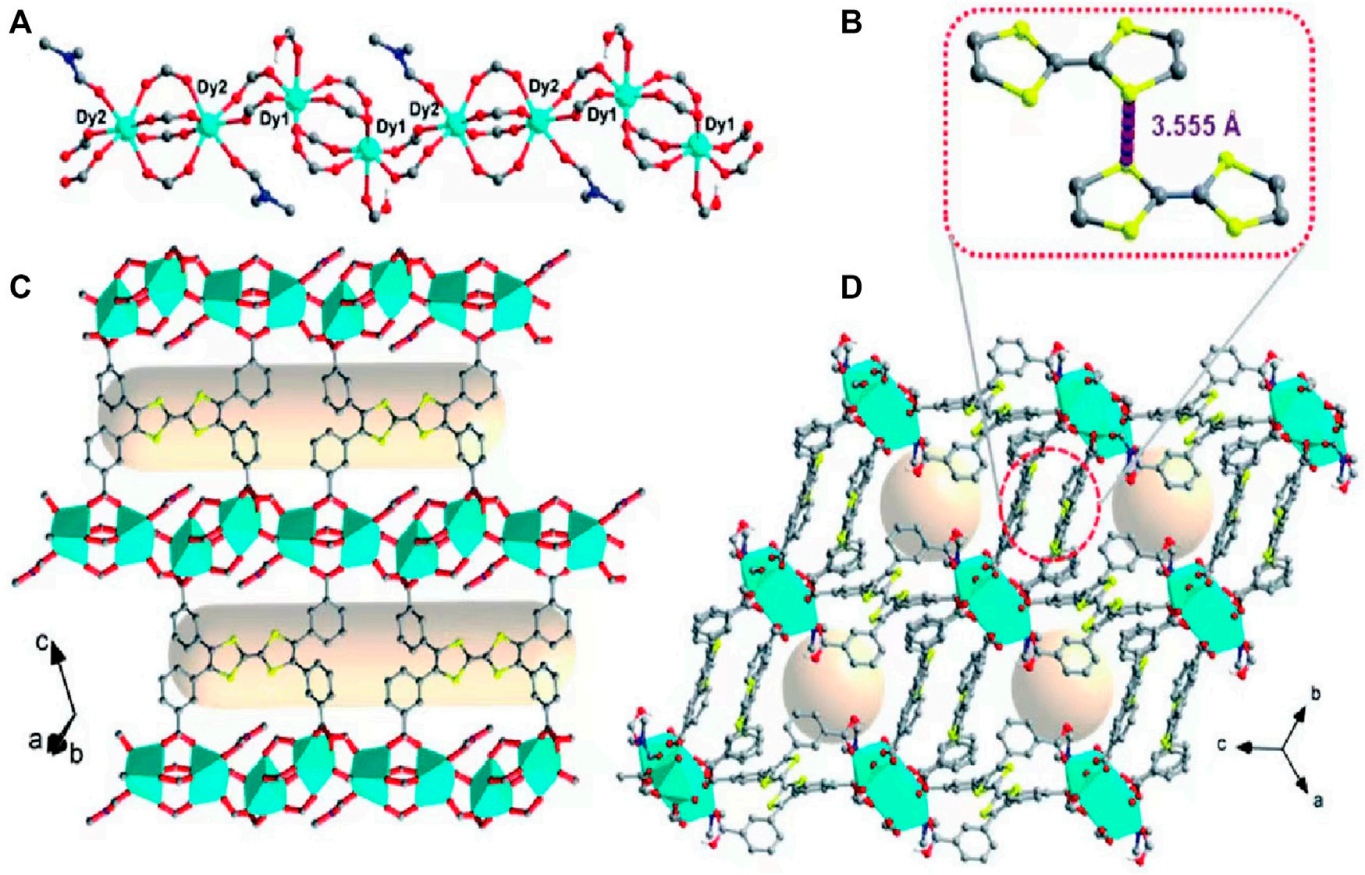
Single-crystal structure of Dy-m-TTFTB. **(A)** The one-dimensional chain of Dy-m-TTFTB; **(B)** the weak interaction between TTF (L1) dimers; **(C)** the two-dimensional structure assembled from the one-dimensional chains and m-TTFTB4- ligands **(D)** view of the three-dimensional framework. Color scheme: Dy, cyan; O, red; N, blue; C, black or gray; S, yellow; H, green. Reproduced with permission from (Su et al., 2022) Copyright (2022) Royal Society of Chemistry.

As one of the most common composite redox nanomaterials, metal-organic framework nanoparticles (MOFs) consist of metal-containing nodes (metal ions or clusters and organic bridging ligands), comprising an attractive class of composite nanomaterials (Zhou et al., 2012; Zhou and Kitagawa, 2014). A large body of literature summarizes the following unique advantages of MOFs as potential drug carriers: highly tunable properties and porous, high drug loading capacity, and controlled versatility (Wang et al., 2018; Zhao et al., 2018; Luo et al., 2019). There has been substantial progress in the design of gated, redox-responsive MOFs as cooperative therapeutic functional drug carriers (Zhou et al., 2021). After reacting m-tetrathiafulvalene tetrabenzoate (*m*-TTFTB) with Dy^3+^ under solvothermal conditions, Su et al. (2022) obtained redox-active MOFs (Ag NPs@Dy-*m*-TTFTB) through post-synthetic redox-tuning and the incorporation of Ag nanoparticles *in situ* for promoting the tuned photothermal NIR response (Figure 4). The results of their *in vitro* experiments showed that, when compared to the control Dy-*m*-TTFTB and I_3_@Dy-*m*-TTFTB, the redox reaction between Ag NPs@Dy-*m*-TTFTB and I_2_ or Ag^+^ significantly improved the absorption of NIR light by generating a stable TTF^•+^ state, which has a high photothermal conversion efficiency. Prussian blue nanoparticles (PBNPs) are unique MOF nanomaterials formed by alternating ferric and ferrous irons coordinated with cyanides (Thompson and Callen, 2004). PBNPs exhibit multienzyme-like activities such as SOD-, CAT-, and peroxidase (POD)-like activities through the abundant redox potentials of their different forms (Liang and Yan, 2019). PBNPs with POD or oxidase activity can generate abundant ROS, while PBNPs with CAT and/or SOD activity can remove ROS (Estelrich and Busquets, 2021). The discovery of this property has led to increased research on the composite redox nanomaterial PBNPs worldwide, especially for redox-based biomedical applications.

## 3 Application of redox nanomaterials in disease therapy

**FIGURE 5:**
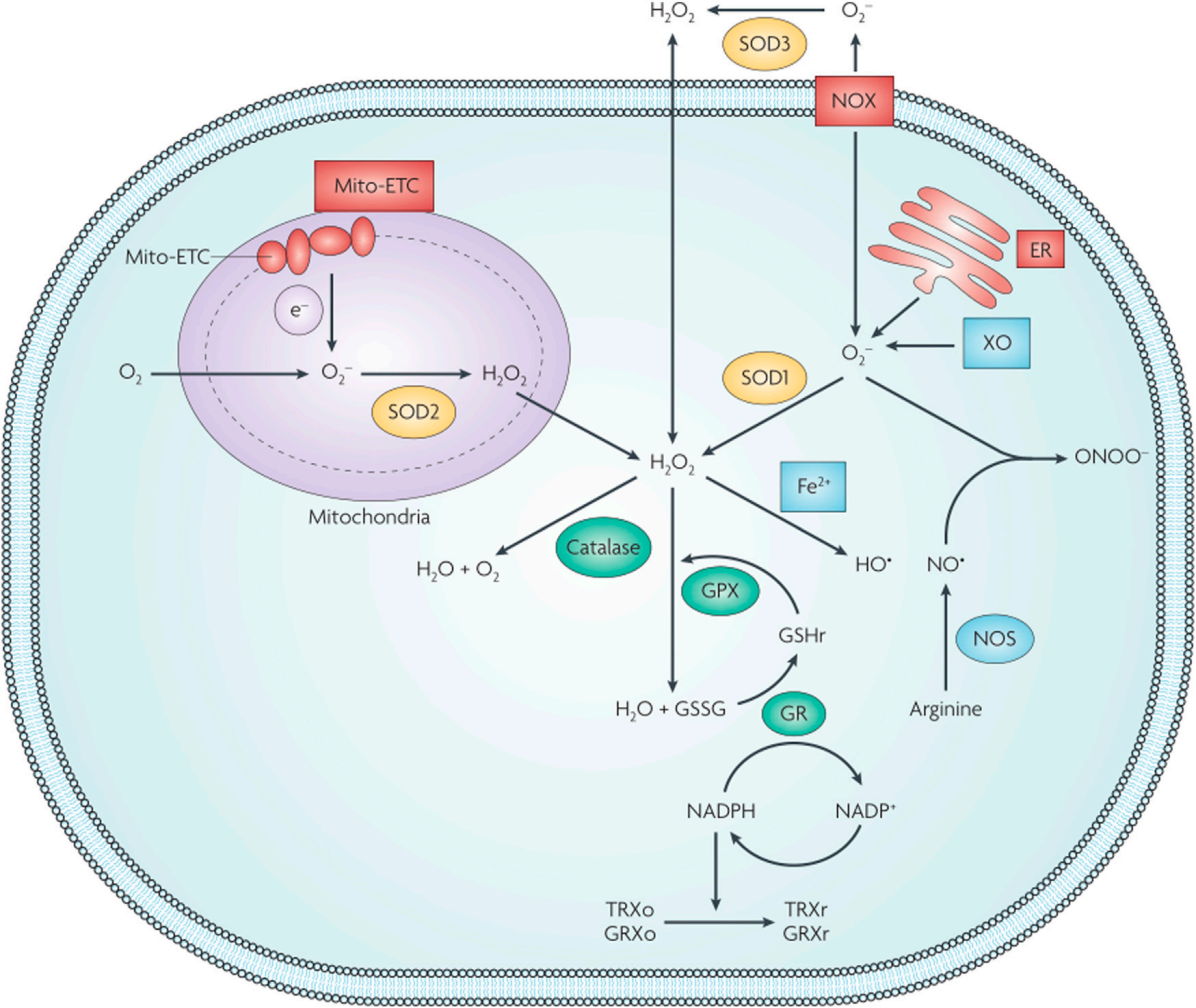
Schematic illustration of cellular redox homeostasis. GPX, glutathione peroxidase; GR, glutathione reductase; GRXo, glutaredoxin (oxidized); GRXr, glutaredoxin (reduced); GSHr, glutathione (reduced); GSSG, glutathione (oxidized); TRXo, thioredoxin (oxidized); TRXr, thioredoxin (reduced); XO, xanthine oxidase. Reproduced with permission from (Trachootham et al., 2009) Copyright (2009) Nature Research.

The dynamic cellular redox balance is maintained by the balance between the production and elimination of ROS (Trachootham et al., 2009) (Figure 5). Some diseases exist in an altered redox state resulting from the buildup of free radicals (such as ROS and H_2_O_2_) or reducing equivalents (such as glutathione and NAD^+^). This observation inspired the development of smart redox-responsive nanomedicines with enhanced ROS-generating/scavenging efficiencies to help maintain the redox balance in different tissues. The functional mechanisms and applications of redox nanomaterials in tumors and other human tissues are briefly discussed.

### 3.1 Application of redox nanomaterials in tumor tissues

Tumor tissues usually include the parenchyma and stroma. The tumor stroma has different morphological characteristics compared to the stroma in normal tissues, and is often referred to as a tumor microenvironment (TME). Due to the abnormal growth of tumor cells and their excessive demand for nutrients, most tumors show dysregulated angiogenesis with leakage of vascular structures, increased stiffness, interstitial pressure, immunosuppression, the overexpression of enzymes, acidity, hypoxia, and an overactivated redox environment (Cheng et al., 2015b; Liu and Huskens, 2015; Tian et al., 2017). These abnormal features in the tumor tissues form a complex intratumoral microenvironment that can promote tumor proliferation and progression (Peng et al., 2019). Moreover, it has become an alternative tumor target in theranostics applications (Khawar et al., 2015).

Evidence has suggested that when compared with normal cells, most cancer cells produce more ROS, and this increase in ROS levels may play an important role in the occurrence and development of cancer (Behrend et al., 2003; Wu, 2006). To balance the oxidative stress caused by ROS overproduction, cancer cells exhibit a high degree of adaptability through multiple pathways, including the recruitment of non-enzymatic ROS scavengers, such as GSH and vitamins C and E (Peng et al., 2019). As a non-protein thiolated antioxidant tripeptide, GSH is widely distributed and has high concentrations in tumor tissues. For example, while the intracellular GSH concentration can reach 10 mM and its extracellular concentration ranges from 2 to 20 μm (Mura et al., 2013), its concentration in tumor tissues is at least four times that of the normal tissues, especially in some multidrug-resistant tumors (Meng et al., 2009; Li and Xie, 2017). Elevated ROS and GSH levels have become characteristics of the TME, making tumor tissues more resistant to chemotherapy (Peng et al., 2019). The differences between the tumor and normal tissue microenvironment also provide a potentially feasible strategy for redox-responsive anti-tumor nanomaterial-based therapeutic systems. Here, ROS-responsive, GSH-responsive, and ROS/GSH dual-responsive anti-tumor nanomaterial therapy systems and their therapeutic mechanisms are briefly summarized.

#### 3.1.1 Reactive oxygen species-responsive anti-tumor nanomaterial therapy systems

High ROS level is a significant histological feature in tumor microenvironment. We can not only use this property as a marker to distinguish tumor tissue from normal tissue, but also kill tumor cells by changing the level of ROS or inducing the production of highly toxic ROS (Huang et al., 2022; Zhang et al., 2022). In addition, it is also one of the mainstream therapeutic strategies to use the high H_2_O_2_ level in tumor microenvironment to catalyze it to produce oxygen and improve the tumor hypoxia environment.

Acidic environments can cause the breakage of some acid-unstable bonds and lead to the disintegration of nanomaterials (Chu et al., 2022). Similarly, in the highly reductive TME, high levels of ROS can also lead to structural changes in chemical bonds or groups; this has become the biochemical basis for tumor-targeted therapeutic strategies (Liu et al., 2016b; Chen et al., 2016). Furthermore, this has promoted the generation of smart “off-on” nanoprobes (Ma et al., 2018), such as the thioketal linker (Yue et al., 2016; Hu et al., 2017), propylene sulfide (Poole et al., 2015), and aryl boronic esters (Broaders et al., 2011). Xu et al. (2017) designed a novel ROS-responsive polyprodrug nanoparticle platform based on the anticancer drug mitoxantrone (MTO) for triggering drug delivery and tumor therapy. As a model drug, MTO can copolymerize with a ROS-cleavable thioketal linker to prepare a polyprodrug. To verify the ROS responsiveness of polyMTO, a control polyMTO without thioketal groups was also synthesized. The results of the gel permeation chromatography assays showed that there was no molecular weight change for the control polyMTO after incubation with the ROS (50 × 10^−3^ M KO_2_ solution) for 24 h. In the experimental group, the relative molecular weight of the polyMTO decreased sharply to 400 g mol^-1^ after being incubated with a KO_2_ solution for 6 h, suggesting that the degradation of polyMTO was based on the ROS-triggered cleavage of the thioketal linker. The *in vitro* and *in vivo* experiments showed that the polyMTO could respond to intracellular ROS in tumor tissues with a chain-breakage patterned release of intact anticancer drugs, thus generating significant inhibition of tumor cell growth.

Tumor cells are reportedly more sensitive to damage caused by exogenous ROS generated after treatment than endogenous ROS generated in the TME (Pelicano et al., 2004). The principle is based on the Fenton reaction or Fenton-like reaction, in which hydrogen peroxide can be converted into highly toxic free radicals under the catalysis of transition metal ions (Gligorovski et al., 2015). The mild acidity and excessive hydrogen peroxide production in the tumor environment provide suitable conditions and reactants for the Fenton reaction (Chen et al., 2021a). Based on this, therapeutic systems for killing tumor cells by inducing ROS overproduction have been widely studied and applied, such as photodynamic (Zhu et al., 2022a; Sun et al., 2022) and chemodynamic therapies (Yao et al., 2021). Qin et al. (2021) assembled lactate oxidase (LOx) and CAT into Fe_3_O_4_ nanoparticles and indocyanine green (ICG) hybrid nanogels (FIGs-LC) to potentially fulfill ROS regulation in tumor tissues. The endogenous ROS content in the tumor tissue cannot generate sufficient free radicals to induce tumor cell apoptosis (Gao et al., 2019). To increase the generation of singlet oxygen and hydroxyl radicals, LOx and CAT were introduced into the nanoplatform, endowing FIGs-LC with peroxisome-like functions. Under the action of both, endogenous lactate and endogenous H_2_O_2_ were selectively converted to ROS or oxygen, thus promoting the oxidative stress response. The anti-tumor experiments showed that FIGs-LC significantly increased the level of intracellular ROS, which caused lethal damage to the tumor cells and effectively inhibited tumor growth.

**FIGURE 6:**
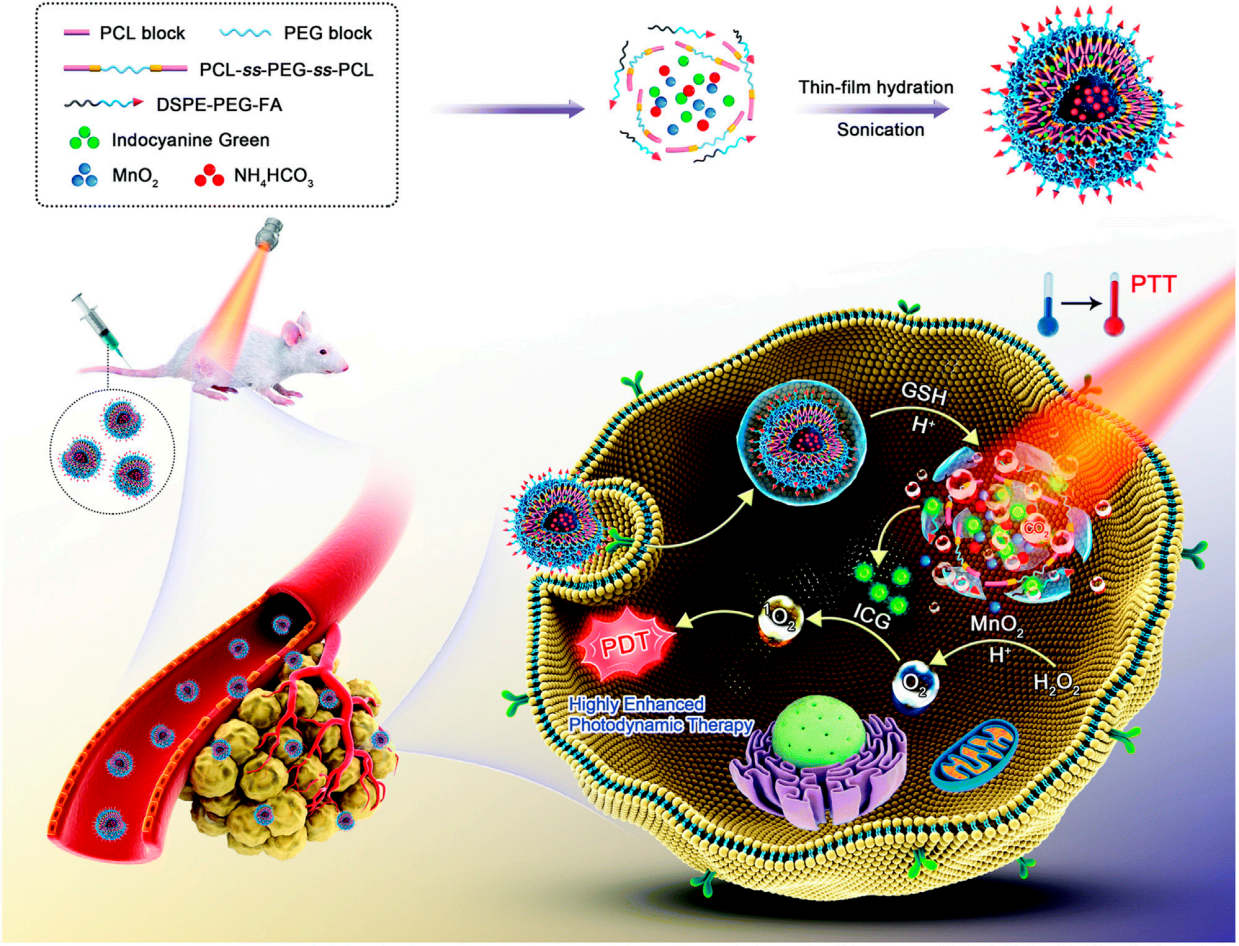
Schematic illustration for the formulation and enhanced photothermal–photodynamic combination therapeutic mechanism of oxygen- and bubble-generating polymersomes (FIMPs). Reproduced with permission from (Li et al., 2021b) Copyright (2021) Royal Society of Chemistry.

As previously noted, in addition to converting hydrogen peroxide into toxic ROS through the Fenton reaction, the high levels of hydrogen peroxide in the TME are often used to generate oxygen *in situ* to improve the anoxic environment of the tumors, thereby inhibiting tumor cell recurrence, invasion, and metastasis (Yu et al., 2018). Hypoxia is also known to increase multidrug resistance to chemotherapy, reduce the sensitivity of tumors to radiation therapy, and lead to reduced benefits for some oxygen-dependent treatments (Tong et al., 2015), such as photodynamic (Cheng et al., 2021) and sonodynamic therapies (Zhang et al., 2021). Nanoplatforms that catalyze the endogenous H_2_O_2_ for *in situ* O _2_ production have thus been extensively studied and applied. Li et al. (2021b) prepared oxygen- and bubble-generating polymersomes nanomaterials (FIMPs), which used MnO_2_ and indocyanine green, the hydrophobic photosensitizer, as the shell and NH_4_HCO_3_ solution as the core, based on the reduction-responsive copolymer (poly (ε-caprolactone)-ss-poly (ethylene glycol)-ss-poly (ε-caprolactone) and 1,2-distearoyl-*sn*-glycero-3-phosphoethanolamine-N-[folate (polyethylene glycol)-2000]), to combine PTT and PDT in tumor treatments (Figure 6). *In vitro* and *in vivo* experiments have shown that FIMPs can facilitate a high accumulation of drugs at the tumor site through EPR and folic acid-mediated targeting effects. At the same time, MnO_2_ wrapped in a hydrophobic membrane can react with endogenous hydrogen peroxide to produce oxygen, which can effectively overcome the hypoxic microenvironment in the tumor and significantly improve the effectiveness of photodynamic and photothermal therapies.

#### 3.1.2 GSH-responsive anti-tumor nanomaterial therapy systems

As one of the most important antioxidants in tumor tissues, the content of GSH is of great significance. Whether through GSH-responsive chemical bonds, direct consumption of GSH (Huang et al., 2021b), or inhibition of GSH production (Zhu et al., 2022b), the basic principle of GSH-responsive anti-tumor nanomaterial therapy systems is to change GSH content.

GSH controls the redox environment of the cell mainly through the formation and breakage of disulfide bonds and reactions with excess ROS (Guo et al., 2018) from a molecular level, which affects the scavenging ROS and helps to maintain a redox balance (Fu et al., 2021). In the TME, nanomaterials with disulfide bonds will disintegrate in a GSH-rich environment to rapidly release drugs or prodrugs, acting as tumor-specific targeted therapies. In addition, as easily modified connectors, disulfide bonds can be attached to the backbones of polymers and used in the conjugation of side chains or nanoparticles with drugs, genes, and targeting groups (Guo et al., 2018). Zou et al. (2020) cross-linked siRNA molecules using disulfide bonds to design a multifunctional siRNA nanocapsule (Ang-NCss(siRNA)) for glioblastoma (GBM) gene therapy. The nanocapsule facilitates blood-brain barrier (BBB) permeation and glioblastoma tissue targeting *via* the receptor-related protein-1 ligand Angiopep-2. The intracellular siRNA rapid release is triggered by an abundance of GSH in the GBM cytoplasm *via* the disulfide bond breakage. The results showed that Ang-NCss (siRNA) exhibited precise tumor targeting, penetration, and efficient and responsive release. An *in vivo* mouse tumor model showed that Ang-NCss(siRNA) had a significant inhibitory effect on U87MG glioblastoma while exhibiting good biocompatibility, indicating that it is a promising, safe, and efficient tool for GBM gene therapy.

As the GSH is overexpressed in the TME, the disulfide bond has been widely used as the “golden standard” for the design of redox-responsive drug delivery systems (Lee et al., 2015; Sun et al., 2018a). In addition to the disulfide bonds, other chemical bonds and groups with GSH responsiveness have attracted research attention, such as the diselenide bond, succinimide - thioether linkage, and trisulfide bond.

**FIGURE 7:**
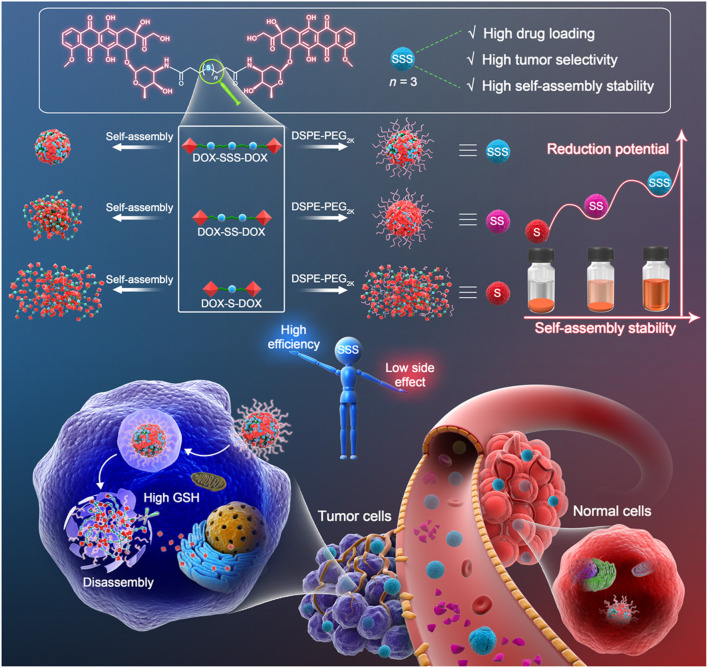
The trisulfide bond–bridged prodrug nanoassemblies for cancer therapy. Simple insertion of a trisulfide bond–transformed DOX homodimeric prodrugs into self-assembled nanomedicines with three highlights: high drug loading, high self-assembly stability, and high tumor selectivity. Reproduced with permission from (Yang et al., 2020b) Copyright (2020) American Association for the Advancement of Science.

As early as 2010, Ma and his colleagues applied diselenide bonds to construct redox-responsive polymer carriers (Ma et al., 2010). The diselenide and disulfide bonds have similar reduction sensitivities and redox abilities (Beld et al., 2007; Zhou et al., 2017). As the energy of the Se-Se bond is lower than that of the S-S bond (Se-Se, 172 kJ/mol; S-S, 268 kJ/mol), the redox property of the drug delivery system based on the diselenide bond may be more sensitive (Zhai et al., 2017). Fang et al. (2020) synthesized programmed drug-releasing nanoparticles (G (TM)PPSP) for TME remodeling and breast cancer therapy, which were synthesized by coupling paclitaxel (PTX) to platinum nanoparticles *via* diselenide bonds, and then coupled with telmisartan (TM)-embedded gelatin nanoparticles. Under the mediated action of the matrix metalloproteinase-2, the gelatin nanoparticles of G (TM)PPSP were degraded in the TME, and TM was released. The remaining portion of the G (TM)PPSP then entered the tumor cells through endocytosis, which disintegrated and released PTX at high GSH levels to induce tumor cell apoptosis combined with the photothermal effects of platinum nanoparticles. Baldwin and Kiick (2013) found that the succinimide bond formed through the Michael-type addition of aromatic thiols to maleimides is redox-sensitive and can be broken *via* GSH cleavage. In addition, when compared to disulfides and other common adducts, the bond expanded the reduction timescale. Oscillatory rheology experiments have also demonstrated the excellent stability of the succinimide–thioether bond. The degradation rate of the succinimide-thioether containing hydrogels was 10 times slower than that of disulfide-crosslinked hydrogels, suggesting that the succinimide-thioether-containing adducts have good blood stability and longer administration times. Compared with the disulfide bond, the trisulfide bond contains an extra sulfur atom, which is the key linkage in biomolecules with functions ranging from subtle modulation of the conformation and assembly stability in proteins and peptides (Bernardes et al., 2007). Yang et al. (2020b) synthesized three kinds of Dox prodrugs using thioether, disulfide, and trisulfide bonds, respectively, and explored the effects of the trisulfide bond on the nano-assembly of homodimer prodrug (Figure 7). High-performance liquid chromatography showed that as the number of redox reaction sites increased, so did the redox potential. Furthermore, the drug release rate of the nanodrug delivery systems containing trisulfide bonds is higher under the same conditions, which shows that the trisulfide bond is more sensitive to redox activity and has great potential as a GSH-responsive bond.

GSH, as an antioxidant, is known to play an important role in maintaining the redox balance in tumor tissues, as high levels of oxidative stress make tumor cells more sensitive to a lack of GSH (Lv et al., 2019). GSH thus not only acts as a stimulus for targeted therapy through responsive, functional nanoparticles, but it can also destroy the redox balance by regulating its content in the tumor tissue, leading to tumor cell death. For example, various metals or metal oxides, while having GSH responsiveness, can also consume GSH in the TME (Wang et al., 2019b). The depletion of GSH means that the destruction of tumor tissue caused by oxidative stress can be expanded to achieve the purpose of the tumor treatment. Xu et al. (2021) synthesized a manganese porphyrin-based metal-organic framework nanosensitizer (Mn-MOF), which was used to enhance sonodynamic therapy and ferroptosis. Manganese porphyrins can not only catalyze hydrogen peroxide with high efficiency through the cyclic conversion of Mn^3+^-porphyrins and Mn^4+^-porphyrins (Lv et al., 2019) but also effectively reduce GSH through its absorption and oxidization (Zhang et al., 2018; Wang et al., 2019a). *In vivo* and *in vitro* experiments showed that Mn-MOF persistently catalyzed the tumor-over expression of H_2_O_2_ so that O_2_ was produced *in situ* to relieve tumor hypoxia and degrade GSH and glutathione peroxidase-4 content, thus promoting oxidative stress and ferroptosis.

#### 3.1.3 Reactive oxygen species/GSH dual-responsive anti-tumor nanomaterial therapy systems

**FIGURE 8:**
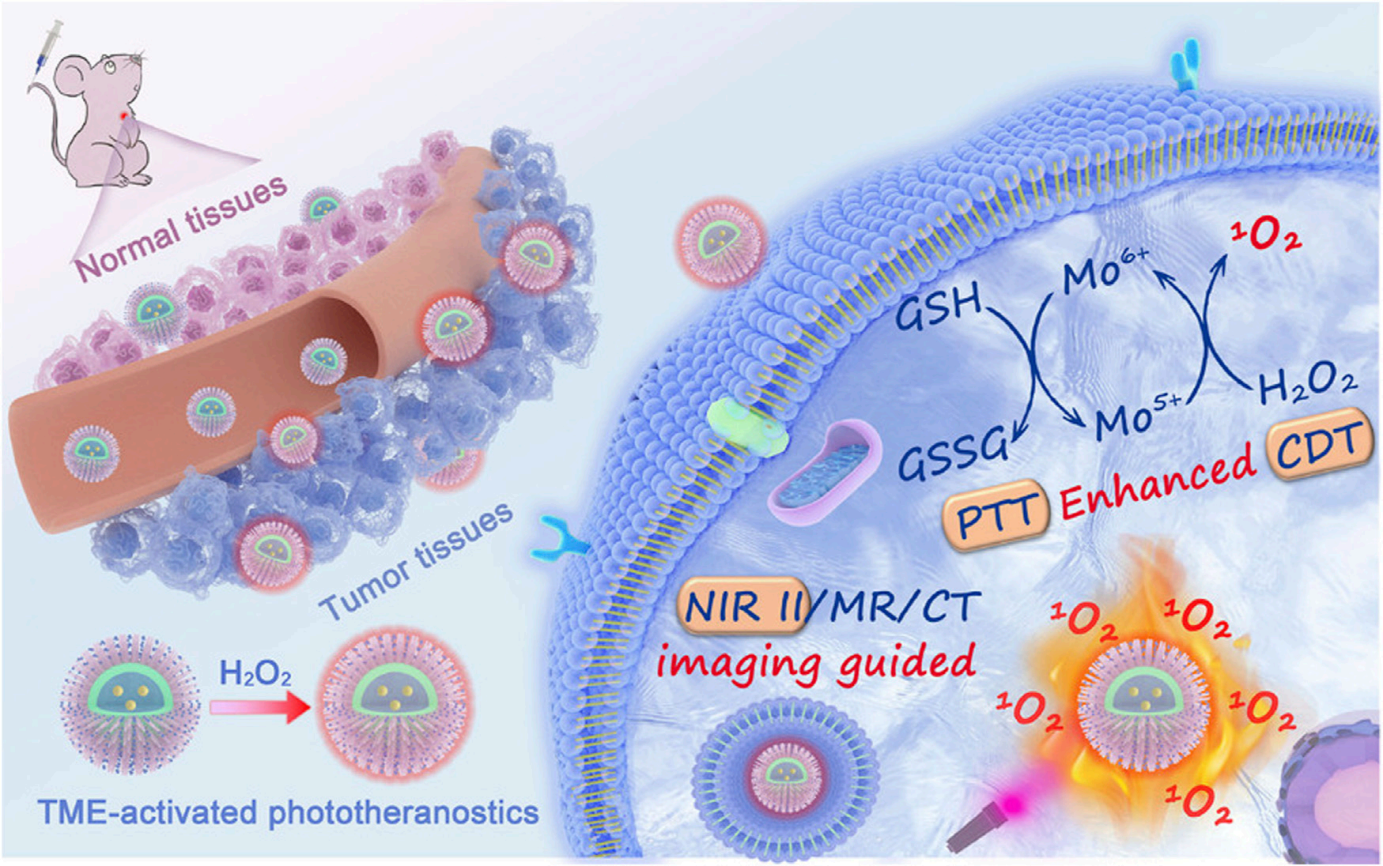
Illustration of the Preparation Procedure and Working Mechanisms of the DCDMs. Reproduced with permission from (Dong et al., 2020b) Copyright (2020) American Chemical Society.

It is not uncommon for nanomaterials to regulate the tumor tissue redox balance through both the ROS and GSH response pathways. Furthermore, their therapeutic mechanisms involve both aspects, such as chemodynamic therapy, which is based on transition metal-based Fenton responses or Fenton-like responses. Many highly valent transition metal ions, such as Mn^4+^ or Cu^2+^, are GSH-responsive but can also decompose endogenous hydrogen peroxide into highly toxic ROS (Ma et al., 2019; Dong et al., 2020a). Compared with the single-pathway response mechanism, the dual-pathway response mechanism works by increasing the level of ROS and consuming GSH, which may be a more effective tumor treatment strategy (Chen et al., 2021a). Dong et al. (2020b) prepared a novel all-in-one theranostic nanoagent (DCDM) (Figure 8), which encapsulated ultrasmall MoO_x_ nanoparticles, a PTT/CDT dual agent, and Nd^3+^-doped down-conversion nanoparticles (DCNPs), a contrast agent, in polyethylene glycol (PEG)-modified dendritic mesoporous silica, for multimodal tumor imaging-guided synergistic therapy. *In vitro* and *in vivo* experiments showed that Mo^5+^ in DCDM can catalyze hydrogen peroxide to produce singlet oxygen according to the Russell mechanism. Subsequently, the generated Mo^6+^ can consume GSH, reduce the GSH level at the tumor site, and weaken the antioxidant capacity of the tumor, thus further accelerating the generation of singlet oxygen and enhancing the efficacy of CDT. Under multimodal *in vivo* imaging guidance, DCDM exhibits superior therapeutic outcomes at a cellular level and possesses remarkable anticancer abilities in U14 tumor-bearing mice.

ROS/GSH double-responsive nanomaterials, which involve the synthesis of two stimulation-responsive nanomaterial therapeutic systems that are modified with responsive functional groups on their surface, have attracted a wide range of research interest. Sun et al. (2021) synthesized a cucurbit [7]urils-based PTX-loaded supramolecular nanomedicine (PTX@PPA-CS NPs) with a GSH-responsive polylactic acid nanoparticle core and non-covalently modified ROS-responsive polymer shell. *In vitro* experiments using 10 mM GSH showed that the PPA-CS NPs exhibited a distinct increase in size over time after incubation with the 100 μM H_2_O_2_ for 19 h. In addition, transmission electron microscopy and mass analysis both confirmed that PA-CS NPs could sequentially respond to high levels of ROS and GSH. Compared with the control group, the tumor tissue in the PTX@PPA-CSNPs group showed the largest necrotic area and the most significant anti-tumor effect.

Although more obvious therapeutic effects may be achieved, the nano-therapeutic platform designed by combining ROS responsiveness and GSH responsiveness will undoubtedly have a more complicated structure, which means more complex synthesis process or higher production cost, and also means that this kind of nanomaterials will face more experimental investigation and difficulties in clinical practice. Therefore, how to balance high efficiency and low price is an inevitable consideration of redox nanomaterials in the field of tumor therapy and even other medical fields.

### 3.2 Application of redox nanomaterials in other tissues

Under certain conditions, subcellular compartments, such as the cytosol in human cells, may have a reducing environment, which could lead to an imbalance in the redox potential in local tissues, such as in inflammation, septicemia, hypoxia, and cancer (Sufi et al., 2019). The fundamental reason for this phenomenon is that cells produce ROS excessively in different subcellular locations, including the mitochondria. Electrons escaping from the mitochondrial respiratory chain can react with O_2_ to generate O_2_^•-^, then be further converted into ROS, such as H_2_O_2_, •OH, and ClO^−^ *via* various catalytic reactions (Sabharwal and Schumacker, 2014). Increased ROS levels can lead to mitochondrial dysfunction, affecting metabolism, reducing ATP production, and further increasing ROS production. This leads to a vicious cycle in the body that can manifest as aging, various diseases such as cardiovascular disease, neurodegenerative diseases, diabetes, and even death (Duchen, 2004; Fink et al., 2014). Numerous studies have shown that applying oxidation-reducing nanomaterials can effectively reduce the production of mitochondrial ROS to promote the mitochondrial redox balance (Wongrakpanich et al., 2014; Xie et al., 2018) and effectively treat disease. The following section contains a brief introduction to how oxidation-reducing nanomaterials regulate the redox balance in tissues associated with brain disease, cardiovascular disease, aging, and other aspects.

#### 3.2.1 Application of redox nanomaterials in brain diseases

Redox activity is an integral part of many metabolic processes nerve cells require to function properly in the brain. ROS generated through intracellular and extracellular reactions are regulators of several signaling pathways involved in various physiological processes (Eleftheriadou et al., 2020). In recent years, a large body of literature has suggested that redox stress, including oxidative and nitrosative, is key for the pathogenesis of brain diseases such as Alzheimer’s disease (AD) (Jiang et al., 2022), Parkinson’s disease (Rizor et al., 2019), traumatic brain injury (Chonpathompikunlert et al., 2012), and stroke (Song et al., 2021). In addition, clinical and animal studies have shown that both oxidative stress and inflammation are involved in secondary brain injury after intracerebral hemorrhage (Wang and Tsirka, 2005; Wang and Doré, 2007).

The structural features of the central nervous system, higher metabolic levels, and lack of antioxidants make it highly susceptible to oxidative stress (Lin and Beal, 2006; Wang and Michaelis, 2010). When ROS or reactive nitrogen species (RNS) diffuse into the intercellular environment of glial cells and neurons, they can cause mitochondrial dysfunction, lipid peroxidation, and DNA damage through oxidative stress and thus induce apoptosis. They can also disrupt the integrity of the BBB through tight junction modifications, matrix metalloproteinase activation, and inflammatory response activation (Song et al., 2020). The therapeutic strategy for loading antioxidants and/or ROS-responsive moieties onto nanocarriers to cross the BBB for treating brain diseases by reducing the production of ROS in neural tissue or scavenging the overproduction of ROS is gaining popularity. Li et al. (2021a) prepared a selenium-peptide nanocomposite (Res@SeNPs) based on resveratrol to improve cognitive disorder in an AD mouse model by decreasing amyloid-β (Aβ) deposition, inhibiting ROS production and neuroinflammation, and regulating the gut microbiota to improve cognitive disorders. The results of their *in vitro* and *in vivo* experiments showed that Res@SeNPs improved the antioxidation status of PC12 cells, inhibiting ROS-induced neuronal cell apoptosis *in vitro*, thus exhibiting superior antioxidation abilities that were effective at reducing oxidative stress *in vivo*.

#### 3.2.2 Application of redox nanomaterials in cardiovascular diseases

Experimental and clinical data show that vascular oxidative stress, which is associated with cardiovascular risk factors such as hypertension (Griendling et al., 2021) and diabetes mellitus (Hink et al., 2001), can predispose patients to atherosclerosis (Sorescu et al., 2002). Atherosclerosis is usually caused by endothelial dysfunction, in which the excessive production of ROS plays a key role (Allahverdian et al., 2014; Allahverdian et al., 2018). Given the problems associated with conventional drug therapy, researchers have been working on developing and designing nanomedicines that can enhance drug delivery and have higher efficacy. Gao et al. (2020) prepared a macrophage membrane-coated ROS-responsive biomimetic nanodrug delivery system (MM-AT-NPs) loaded with the model drug atorvastatin (AT) for the treatment of atherosclerosis. *In vivo* studies have shown that MM-AT-NPs target inflammatory tissues through encapsulated macrophage membranes and that AT is released from the MM-AT-NPs upon stimulation by high levels of ROS, thus reducing atherosclerosis by decreasing the number of perivascular CD31^+^ neovasculature and inhibiting KI67^+^ endothelial cell proliferation.

Coronary heart disease, including acute myocardial infarction, blockage of the coronary artery, and cardiac failure, is one of the leading causes of death worldwide (Li et al., 2022). In patients with acute myocardial infarction, restoring coronary blood flow (reperfusion) through medications and/or revascularization procedures is essential (Vicencio et al., 2015). However, during reperfusion, a vicious cycle between the sudden release of ROS and apoptosis of cardiomyocytes can lead to myocardial ischemia/reperfusion injury (MI/RI) (Hausenloy and Yellon, 2013). To address the need for efficient drug transport to the ischemic myocardium and to increase drug accumulation, attention has turned to functional nanodrug carriers. Li et al. (2022) used the ability of platelets and regulatory T cells (Tregs) to actively target the ischemic myocardium to synthesize platelet membrane-encapsulated regulatory T cell biomimetic nanoparticles (CsA@PPTK) loaded with cyclosporine A (CsA) for the treatment of MI/RI. Among them, poly (5,5-dimethyl-4,6-dithiopropylene glycol azelate) (PTK) contains a large number of thioketal linkers, which can effectively scavenge high levels of ROS from the ischemic myocardial microenvironment (Shim and Xia, 2013). The results of the drug release experiments, *in vitro* cellular assays, and *in vivo* experiments all showed that CsA@PPTK not only releases CsA in response to high ROS levels in the ischemic myocardial microenvironment but that it also has excellent ROS scavenging ability, resulting in a high survival rate in H9C2 cells.

The strategy for modulating or scavenging ROS in diseased tissues through the construction of redox nanomaterials has been widely applied in treating cardiovascular disease. Yang et al. (2019) summarized the therapeutic mechanisms of these nanomedicines in cardiovascular diseases, which mainly include the following two pathways: avoiding cellular damage from oxidative stress by isolating the ROS microenvironment and reducing oxidative stress-induced dysfunction in tissues or organs by lowering ROS levels. This also provides directions for future nanomaterial design, such as designing a dual-pathway nanomedicine to regulate redox homeostasis in tissues to achieve more desirable therapeutic effects.

#### 3.2.3 Application of redox nanomaterials in anti-aging

Researchers have hypothesized that the characteristic changes that occur with aging result from the accumulation of random oxidative damage to cellular molecules caused by oxygen and nitrogen-based radicals produced under normal physiological conditions (Harman, 1956). Enhanced resistance to oxidative damage may also lead to an increase in lifespan (Harman, 1981; Larsen, 1993). This gave rise to the idea that nanomaterials with excellent reducing properties could help to protect the body from oxidative stress damage by scavenging ROS (Mao and Cai, 2021). Among the materials, there have been exciting results in the design and study of fullerenes (Galvan et al., 2017) and cerium oxide (Heckert et al., 2008), which can reduce the damage caused by ROS and nitrogen species.

Fullerenes were first shown to compete with radical spin traps for both •OH and O_2_^•-^, which were discovered by Dugan et al. using electron paramagnetic resonance techniques (Dugan et al., 1997). There have consequently been numerous studies on fullerenes that can effectively blunt ROS-dependent mediated biological injury (Karakoti et al., 2010). Cong et al. (2015) used *Caenorhabditis elegans* as a model organism to evaluate the potential influence of fullerenol on aging and stress resistance. Under normal conditions, fullerenol induced delayed aging in worms without biological toxicity. Under oxidative stress, fullerenol displayed distinct anti-stress effects not only by upregulating the expression of several anti-stress genes but also *via* ROS scavenging *in vivo*.

It is found that cerium oxide nanoparticles by virtue of the surface Ce(III)/Ce(IV) valency switch provides ROS scavenging abilities (Walkey et al., 2015). This ability has been demonstrated in various disease models (Lord et al., 2021) and explored extensively for various biomedical applications. Similarly, single-walled carbon nanotubes could inhibit telomerase activity by selectively stabilizing human telomeric i-motif DNA (Zhao et al., 2008; Chen et al., 2012). Del Turco et al. (2019) evaluated the anti-senescence activity of cerium oxide nanoparticles (NC) on human endothelial cells by measuring the changes in telomere length. The *in vitro* results showed that the NC inhibited H_2_O_2_-induced telomere shortening in endothelial cells by reducing oxidative stress and scavenging ROS, exhibiting anti-premature senescence properties.

#### 3.2.4 Application of redox nanomaterials in other diseases

Previously, we mentioned that mitochondria are the main organelles that produce ROS and that large amounts of ROS can lead to mitochondrial dysfunction, disrupting cellular function. Noteworthy, mitochondria are also functional organelles for intracellular lipid metabolism, whose damage and reduction in the number can affect lipolysis, leading to lipid accumulation, insulin resistance, and eventually, obesity (Li et al., 2019b). Numerous studies have also confirmed that with obesity, overstuffed adipose tissue produces large amounts of ROS (Hotamisligil, 2006). Therefore, the application of antioxidants to scavenge ROS, reduce oxidative stress, and alleviate fat accumulation and metabolic disorders is thus currently one of the primary options for treating obesity (Xiao et al., 2011). Nanomedicines with antioxidant effects have been widely used in the treatment of obesity or its related diseases, such as metal oxide nanoparticles (e.g., cerium oxide nanoparticles) (Rocca et al., 2015; Nguyen et al., 2016), precious metal nanomaterials (e.g., silver nanoparticles) (Thanh and Green, 2010; Yue et al., 2019), and organic nanomaterials (e.g., Coenzyme Q10) (Sharma et al., 2012). Non-alcoholic fatty liver disease (NAFLD) is closely associated with obesity (Wree et al., 2013). It has been found that oxidative stress due to increased ROS plays a central role in the progression of disease inflammation and fibrosis in NAFLD (Koek et al., 2011). Eguchi et al. (2015) synthesized redox polymer micelle nanoparticles (RNPs) based on poly (ethylene glycol)-b-poly [4-(2,2,6,6-tetramethylpiperidine-1-oxyl) aminomethylstyrene, which possessed antioxidant nitroxide radicals in the hydrophobic segments. Results from *in vivo* experiments showed that RNPs maintained normal redox reactions in healthy cells; however, they could reduce oxidative stress significantly in liver tissue and promote improvement in liver fibrosis and inflammation upon delivery to the liver tissue.

As well as treating obesity and related diseases, redox nanomaterials have also been shown to have promising antibacterial effects. Undoubtedly, diseases caused by various pathogenic bacteria have historically posed serious global challenges threatening public health (Hu et al., 2018). Antibiotics have been widely used in bacterial infections for over 70 years, while their misuse enables bacteria to evolve quickly and develop resistance (Collignon et al., 2018). As the link between nanotechnology and biomedicine continues to deepen, nano-antimicrobials that have intrinsic antimicrobial activities or potentiate the efficacy and safety of conventional antimicrobial drugs (Ali et al., 2020) can not only have better antimicrobial therapeutic efficacy with fewer side effects but can also control drug release, which has advantages over conventional drugs in terms of circulation and targeted delivery (Jamil et al., 2017). Some metal nanoparticles, such as zinc oxide, can kill intracellular bacteria in macrophages by inducing ROS and NO production (Pati et al., 2014).

Overall, the balance between oxidants and antioxidants are essential for mechanisms related to biological growth, health, and aging, and they are vital for the physiological processes that allow cells to function properly. Whether it is a tumor or other tissues or organs in the body, an imbalance in the redox state within a cell or tissue can lead to various malfunctions in its internal metabolism. Due to the rapid development of nanotechnology, scholars have explored various redox nanomaterials with unique properties to influence redox homeostasis in different tissues (Yang et al., 2019). For example, in the field of anti-tumor therapy where redox nanomaterials are widely used, ROS-responsive, GSH-responsive and ROS/GSH dual-responsive nanomaterials play an excellent therapeutic role with their unique characteristics. In addition, redox nanomaterials also have unparalleled potential and advantages in the treatment of diseases in other human tissues, such as stroke, neurodegenerative diseases, atherosclerosis, obesity, aging and so on. However, despite the widespread use of nanomaterials, we cannot yet precisely define the toxicity towards humans and surrounding biota (Zuberek and Grzelak, 2018). Nanoparticle-induced toxicological mechanisms have been a hot topic in toxicological research and debate in recent years (Marano et al., 2011). The paradigm of the central role of oxidative stress in cellular responses has been suggested as the main explanation for the toxicity of NPs (Nel et al., 2006), which is closely related to the chemical compositions of these NPs and the interactions with the cellular components (Koike and Kobayashi, 2006). However, how to balance the redox equilibrium in diseased tissues with the toxicity of the nanomaterials needs to be explored in greater detail.

## 4 Summary and future perspectives

In this review, we have briefly summarized the various redox nanomaterials used in biomedical applications and grouped them into three categories according to their structural composition, namely: inorganic, organic, and composite. Furthermore, in the field of tumor therapy, where redox nanomaterials are most widely used, three types of redox nanomaterials were identified: ROS-responsive, GSH-responsive, and ROS/GSH dual-responsive. The applications of these materials are described according to their therapeutic principles. In addition, the application of redox nanomaterials in other human tissues in relation to brain diseases, cardiovascular diseases, anti-aging, antibacterial, and obesity, was briefly reported.

Although redox nanomaterials are widely used for the prevention and treatment of various diseases in the preclinical stage due to their advantages, clinical applications are still rare. Most research on redox nanomaterials has focused on *in vitro* and xenotransplantation-based animal experiments. However, these model experiments are different from actual tumors or other pathological changes, especially metastatic tumors, and it is difficult to accurately imitate the damage to cells or tissues caused by various oxidative stress conditions in the human body. The lack of demonstration in clinical trial results is the key factor limiting the clinical application of redox nanomaterials. In the following statements, we briefly analyzed why it is difficult to apply redox nanomaterials to clinical experiments and provided future perspectives on redox nanomaterials.

1) Considering the complexity of the human internal environment, the current evaluation methods for the biocompatibility of different redox nanomaterials are insufficient. Understanding the specific toxicity mechanisms of redox nanomaterials and improving the detection methods of cytotoxicity *in vivo* comprises the basis for biocompatibility evaluations. At the same time, in order to establish a mature evaluation index of biocompatibility, not only systemic toxicity should be tested, focusing on evaluating the retention of nanomaterials in important organs such as the liver and kidney, but also the corresponding human cell lines should also be established to evaluate biosafety *in vitro* in different disease tissues and promote the development of redox nanomaterials in clinical trials.

2) Although redox nanodrugs lasts longer than traditional drugs and the side effects is smaller, their overall therapeutic effects are currently too low to be extended to large-scale clinical applications. In addition, the design cost for the complex structures of the redox nanodrugs, especially the multifunctional nanoplatform therapy system, is too high to be suitable for a wide range of applications. When designing redox nanodrugs, materials that are easy to synthesize and have a low cost should be selected. Meanwhile, it is necessary to adopt simple and effective synthesis methods, and rationally construct composite redox nanomaterials to achieve higher therapeutic effect.

3) Finally, the literature has widely shown that redox nanomaterials with stronger permeability and retention effects tend to have drug delivery efficiencies of ≤5%. Therefore, efforts to eliminate pre-leakage and improve the precise drug-controlled release ability of redox nanomaterials in time and space must be made.

We believe that as nanotechnology and biomedicine are rapidly developing, redox-responsive nanomaterials will gradually overcome these problems and become more applicable in clinical medicine in the future.
